# The Role of CD4+ T Follicular Helper Cells in HIV Infection: From the Germinal Center to the Periphery

**DOI:** 10.3389/fimmu.2017.00046

**Published:** 2017-01-30

**Authors:** John Patrick Thornhill, Sarah Fidler, Paul Klenerman, John Frater, Chansavath Phetsouphanh

**Affiliations:** ^1^Imperial College London, London, UK; ^2^University of Oxford, Oxford, UK

**Keywords:** HIV, Tfh cells, reservoir, T cells, viral immunity

## Abstract

T follicular helper cells (TFh) are key components of the adaptive immune system; they are primarily found in germinal centers (GCs) where their interaction with B cells supports humoral immune responses and efficient antibody production. They are defined by the expression of CXC receptor 5, program death-1, ICOS, and secretion of IL-21. Their differentiation is regulated by B-cell lymphoma 6. The relationship and function of circulating TFh to *bona fide* TFh resident in the GC is much debated. HIV infection impacts the TFh response with evidence of aberrant TFh function observed in acute and chronic infection. Effective TFh responses are associated with the development of broadly neutralizing antibody responses to HIV and may be important for viral control. In addition, TFh are preferentially infected and act as a key reservoir for latent HIV infection. This review explores recent developments in our understanding of TFh differentiation, regulation, function, and the relationship between cTFh and those in GCs, and the complex interaction between TFh and HIV infection.

## Introduction

Optimal immune function requires effective communication between all arms of the immune system. An efficient humoral immune response to pathogens is dependent on the interaction between helper T cells and B cells. T cell help is critical to optimize antibody production and class switching and allows for the development and production of targeted high-affinity antibodies. The subset of T helper cells responsible for this interaction remained elusive until the beginning of this century when Kim et al. described a subset of CXC receptor 5 (CXCR5)+ T cells in the germinal center (GC) with B helper activity (1) coined T follicular helper cells (TFh). TFh cells play a significant role in supporting B cell activation and antibody production during the humoral immune response. They are critical for B cell support, somatic hypermutation (SHM), and antibody class switching (2). TFh dysfunction and expansion has been implicated in a number of disease states including Rheumatoid Arthritis (3), SLE (4), and other autoimmune diseases.

In health, the interaction between Tfh, B cells, and IL-21 supports B cells to proliferate and differentiate into plasma cells thereby leading to efficient antibody production. Consequently, the relationship between TFh cells, humoral immunity, and mechanisms for viral persistence in the setting of chronic viral infections has received much attention. The role of TFh in supporting specific antibody responses has been described in hepatitis C (5), hepatitis B (6), and lymphocytic choriomeningitis virus (LCMV) clearance in mice (7). In acute hepatitis C, the development of HCV-specific antibodies correlates with increased ICOS expression (5), while increased frequencies of TFh is associated with an active Hepatitis B phenotype (6). In the murine model, epigenetic changes which support TFh differentiation have been shown to be important for resolution of LCMV infections (7). These observations support the role of TFh cells in the development of effective pathogen-specific antibody responses to chronic viral infections. The interplay between HIV infection and TFh cells is complex, with evidence supporting dysregulation of TFh function by HIV itself and conversely, that the frequency of TFh subsets positively correlates with effective humoral responses to HIV, as measured by the development broadly neutralizing antibodies (BNAbs) (8). This review aims to summarize the current data and recent advances in our understanding of the role of TFh in HIV infection, with particular emphasis on the phenotypic and functional differences between GC and peripheral-circulating TFh cells (pTFh).

## Phenotypic and Transcriptional Profile of TFh Cells

**Figure 1:**
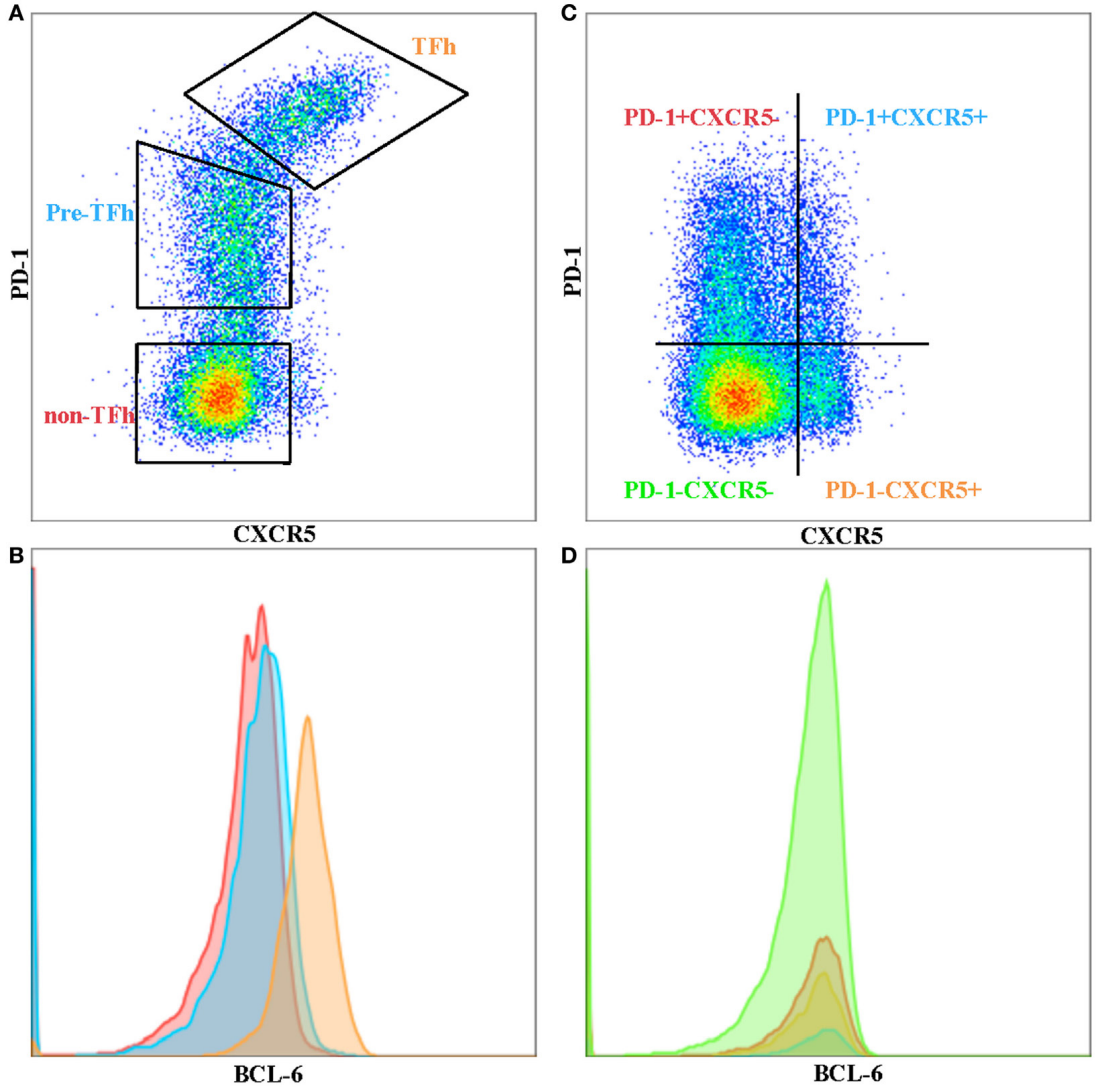
**Phenotypic differences between tonsil and peripheral blood T follicular helper cells (TFh)**. **(A)** TFh, pre-Tfh, and non-TFh subsets in human tonsil. **(B)** BCL-6 expression on TFh (orange), pre-TFh (blue), and non-TFh (red) subsets in tonsil. **(C)** *Ex vivo* peripheral blood CD4 T cells separated into four quadrants comprising program death-1 (PD-1)− CXC receptor 5 (CXCR5)−, PD-1+ CXCR5−, PD-1+ CXCR5+ and PD-1− CXCR5+ subsets. **(D)** BCL-6 expression on PD-1− CXCR5− (green), PD-1+ CXCR5− (red), PD-1+ CXCR5+ (blue), and PD-1-CXCR5+ (orange) subsets in peripheral blood.

T follicular helper cells are a subset of memory CD4 T cells that are localized in the B-cell follicles of secondary lymphoid tissues and provide help for B cells. TFh–B cell interactions allow the production of high affinity, class-switched antibodies following natural infection or vaccination, as well as the establishment of B cell memory (9). TFh cells are defined by their expression of high levels of surface markers of program death-1 (PD-1) and chemokine CXCR5, especially within the GCs of secondary lymphoid tissue. PD-1+ CXCR5+ phenotypically distinguishes GC T-follicular helper cells from other T helper cell subsets and from peripheral CXCR5+ cells with the capacity for B cell help (Figure 1A). This phenotype holds true for GC TFh cells, but peripheral-circulating TFh cells seem to have lower levels of PD-1, where a considerable proportion of CD4+ T cells express only CXCR5 (Figure 1B). However, TFh cells’ identity as a “*bona fide”* subset of T helper cells was not established until B-cell lymphoma 6 (Bcl-6) was discovered to be the “master” transcription factor that drives TFh cell differentiation and function (10–12).

In the early 2000s, several groups described CXCR5+ T cells as having the preferential ability to activate B cells to produce class-switched antibodies (1, 13). The chemokine receptor CXCR5 plays an important role in promoting T cell and B cell migration into B cell follicles in response to its interactions with CXCL13 (14). It has since been shown that these CXCR5+ T cells are TFh cells with a unique gene-expression profile compared to other CD4 T cell subsets. TFh cells do not express Tbet, GATA3, RORyt, or Foxp3 and produce limited Th1/Th2/Th17 related cytokines. Gene-expression profiling of CXCR5+ TFh cells identified key molecules, i.e., *BCL6, ASCL2, IL21, PDCD1* (*PD-1*), and *ICOS*, all of which are involved in the development, migration, and function of these cells (15, 16).

Naïve CD4 T cell differentiation toward the TFh lineage is primed by antigen-presenting dendritic cells (DCs) in the T cell zone, through the interaction between T cell receptor and major histocompatibility complex class II, and costimulatory molecules CD28 and ICOS. Activated antigen-specific CD4 T cells downregulate CCR7 and upregulate CXCR5, allowing them to migrate to B cell follicles. It has recently been shown that the initial step in TFh induction is the upregulation of aschaete-scute homolog 2 (Ascl2), which is a transcription factor that can induce CXCR5 expression, enabling pre-TFh cells to migrate toward the border of the B-cell follicle. Ascl2 has also been shown to repress non-TFh genes, allowing pre-TFh to differentiate into the follicular pathway (17).

Dendritic cells and B cells collaborate in TFh induction in a sequential manner, whereby TFh priming by DCs occurs prior to B cell presentation, allowing for optimal TFh development and GC formation (18, 19). DCs also secrete cytokines IL-6 and IL-12, which induces an early wave of Bcl-6 expression in pre-TFh cells, in a STAT3-dependent manner (20, 21). pre-TFh cells that receives a second signal from cognate antigen-primed B cells stabilizes Bcl-6 express (22). Bcl-6 corroborates with other transcription factors such as, IRF4, BATF and c-Maf, to drive the expression of Tfh cell signature genes critical for T cell–B cell interaction, which includes *CXCR5, ICOS, Sh2d1a Pdcd1*, and *CD40L* (11). IL-27 can induce c-maf expression in collaboration with ICOS (23, 24). C-maf induces IL-21 production in CD4 T cells committed to TFh pathway. IL-21 acts as an autocrine cytokine to promote pre-TFh cell differentiation and homeostatic maintenance of TFh cells. It also plays a vital role in differentiation of GC B cells into memory and plasma cells. Optimal interaction between TFh and B cells determines the magnitude of the GC reaction and somatic mutation that in turn controls affinity maturation of B cells and, therefore, the breadth of the antibody response (25–27).

## Peripheral-Circulating and GC TFh Cells

CXC receptor 5+ circulating TFh (pTFh)-like cells can also be found in human peripheral blood. However, the phenotype of pTFh is not as “clear-cut” compared to GC TFh. They are generally defined as being CXCR5+, CCR7lo, PD-1+, and ICOS+, although this phenotype is not as stringent as GC TFh (28). Others have reported pTFh to co-express CCR7 and are included in the central memory subset. It has also been shown that CXCR5+ Tcm cells lack CXCR3 and CCR4 and do not differentiate into Th1 or Th2 cells upon polarizing cytokine stimulation (2, 29). CXCR5 and PD-1 are stably expressed on pTFh cells and are not transiently upregulated upon non-specific antigen or cytokine stimulation. pTFh expressing these markers can help B-cells to differentiate into plasmablasts, but require secondary signals from B-cells, such as CD40L or ICOS interactions, as well as IL-21 secretion (8, 30, 31). However, blood pTFh and lymphoid TFh cells are clearly phenotypically different, particularly with respect to the expression of PD-1 and BCL-6 (32). Bcl-6 expression can be used to determine TFh in GCs (Figure 1B), but this is not the case in peripheral blood (Figure 1D), where Bcl-6 seem to be downregulated in *ex vivo* CD4 T cells. CXCR5+ PD-1+ pTFh subset contains higher *IL21* mRNA compared to the other CD4+ subsets (Figure 1C) (unpublished data), with CXCR5+ PD-1− subset expressing higher levels of *ICOS*. It remains to be seen whether the CXCR5+ PD-1− subset are precursors to GC TFh or TFh cells that have lost PD-1 expression in the periphery. Iyer and colleagues have shown that blood TFh may be a potential surrogate for GC responses by using a Rhesus macaque model (33). They sampled blood and lymph node at 1 and 2 weeks following SIV booster immunization and demonstrated that blood TFh effector responses (identified by Ki-67+ CXCR5+ expression) predicted magnitude of subsequent GC responses. Further studies in different disease contexts are required to correlate the phenotypic and functional differences previously observed between pTFh and GC TFh in order to understand the important dynamics of this subset in blood and tissue.

## HIV and TFh Dysfunction

The impact of HIV on the frequency and function of TFh subsets during both treated and untreated HIV infection is poorly understood. Host, viral, and treatment factors, such as the timing of Antiretroviral Therapy (ART) initiation with respect to stage of HIV infection duration, may influence the ability of TFh cells to support effective humoral responses. Furthermore, the significance of pTFh and their relationship to GC TFh cells is much debated in health (34) and is likely to be further complicated by chronic HIV infection.

Untreated chronic HIV infection results in selective accumulation of TFh cells in lymph nodes (35); Lindqvist et al. have demonstrated the relative accumulation of HIV-specific TFh cells in lymph nodes during chronic untreated HIV infection that was associated with HIV viremia, interestingly the majority of the expanded TFh cells were specific for Gag, as opposed to Env (36). The expansion of TFh is in contrast to the expected depletion of activated total CD4 T cells seen in untreated HIV infection. The observed TFh expansion is thought to be driven by persistent antigenic stimulation mediated in part by IL-6 signaling (37) which in turn drives the abnormal expansion of TFh cells, skewed B cell differentiation and impaired antibody production (35). Work by Cubas et al. support these findings and suggest PD-1 triggering by PD-L1 on Germial Center B cells as a mechanism for the observed abberant TFh dysfunction (38); their work demonstrates that PD-1 blockade enhances HIV-specific immunoglobulin production *in vitro* and that triggering of PD-1 on TFh reduces cell proliferation and activation, in addition to a decrease in IL-21 production (38). IL-21 has been shown to be essential for provision of B cell help by TFh (30), and its addition rescues antibody production *in vitro* (37). More recent evidence suggest that the loss of regulatory control by T follicular regulatory (TFR) cells may also play a role in the inefficient GC responses seen in untreated HIV infection (39). Data from Rhesus Macques (RM) indicate that TFR may contribute to the regulation of TFh and GC B cells and that decreases in the TFR/TFh ratio during chronic SIV infection may lead to an uncontrolled expansion of both TFh and GC B cells (40).

The difficultly in sampling the GC TFh population has prompted investigation of pTfh as a surrogate marker for GC Tfh activity. Studies have demonstrated the ability of pTfh to provide B cell help (30, 41, 42). Work by Boswell et al. showed differential cytokine production by pTFh (such as IL-21, IL-2, and IL-17) compared with germinal Tfh (IL-4, IL-10, and IL-21, but compromised production of IL-2 and IL-17) (41), while Locci et al. have found that PD-1+ CXCR3− CXCR5+ CD4+ T cells were the subset with greatest ability to provide B cell help (8). Contrary to the expansion of GC TFh seen in chronic HIV, a study of pTFh reported a significant decrease in the pTFh population from HIV-infected subjects compared to HIV-uninfected subjects, however this study did not involve concurrent sampling of GC Tfh (41). In addition, pTfh of chronic ART-treated aviremic individuals have been found to be functionally impaired in their ability to provide adequate B cell help when compared with those from elite controllers (43), while vaccine-induced Ab response to influenza has been associated with preserved Tfh function as measured by the secretion of IL-21 and CXCL13 (44). A recent study by Schultz et al. used an IL-21 capture assay to identify circulating IL-21+ CD4+ T cells. These were found to be transcriptionally and phenotypically pTFh cells (45). In addition to B cell help, IL-21 signaling also supports the antiviral function of CD8 and NK cells thereby playing an important role in control of chronic viral infections (46, 47). Increases in IL-21-producing blood CD4+ T cells have been observed during acute and chronic HIV infection with the elevated frequencies of HIV-specific IL-21-producing CD4+ T cells associated with viral control (48). An expansion of HIV-specific IL-21+ pTFh cells in the ALVAC+ AIDSVAX vaccine study supports the hypothesis that induction of TFh cells might be involved in the superior humoral response (45). Taken together, these data seem to suggest that, in some individuals, HIV infection depletes pTFh numbers and impairs their function, while those better able to maintain robust TFh functionality may have greater propensity for viral control; however, lack of a standardized phenotypic definition for pTFh may confuse some of the reported findings. Furthermore, TFh function may be impacted by ART; initiation of ART early or during Primary HIV infection has been shown to be associated with later posttreatment viral control (49); a recent study investigated the impact of ART in acute HIV infection on TFh function report that early ART may prevent immune dysregulation while preserving pTFh function and B-cell memory, however, the benefit was less if ART is commenced in later stages of acute infection (36).

## TFh, Broadly Neutralizing Antibodies, and HIV Control

Hypergammaglobulinemia is a feature of untreated HIV infection and has been linked to an aberrant TFh–B cell interaction (37, 50), studies have shown a correlation between the expression of Bcl-6 in TFh cells and levels of total serum IgG antibody levels in untreated HIV (36). In the LCMV model, a model for chronic viral infection, the expanded TFh population has been shown to activate non-specific B cells resulting in hypergammaglobulinemia. This has also been observed during other persistent viral infections including HIV and hepatitis C (51).

The generation of BNAbs against HIV is observed in up to 20% of HIV-positive individuals (52). BNAbs are characterized by high levels of SHM which is supported by TFh during the GC reaction (53). Cohen et al. have shown an association between the frequency of PD-1+ CXCR5+ CD4+ Tfh cells during early-untreated HIV infection and future BNAbs development (54). A separate study has shown that the frequency of pTFh cells or more specifically PD-1+ CXCR3− CXCR5+ CD4+ T cells correlate with broadly neutralizing antibody responses (5). A longitudinal study using RMs found that high levels of continuous *Env* antigen production are required for driving GC TFh activation, which in turn leads to more effective broadly neutralizing antibody responses (55). In that study, Env-specific TFh cells but not total TFh correlated with IgG+ GC B cells and effective antibody production (55). These data imply that a period of uncontrolled viral replication (or adequate vaccination) may be required for a sufficient level of antigen exposure and subsequent effective BNAbs production.

The role of immune tolerance has also been implicated in the development of BNAbs, work by Moody et al., show that HIV-1-infected individuals with BNAbs had a higher frequency of blood autoantibodies, a lower frequency of regulatory CD4+ T cells, a higher frequency of memory pTFh, and a higher TFR PD-1 expression compared with HIV-1-infected individuals without BNAbs. The authors suggest the balance of TFR to TFh may allow for development of BNAbs (56).

## TFh Support HIV Persistence

Effective ART reduces plasma viral replication to undetectable levels (as measured by conventional HIV RNA qPCR assays to <20 copies HIV RNA/ml). However, complete eradication of HIV is still unachievable due to low-level viral replication in sanctuary sites and reactivation of virus from the latent HIV DNA reservoir. Suppression of HIV viremia in peripheral blood does not necessarily reflect viral suppression in lymphoid tissue with variable tissue penetration of ART (57), further potentially skewing the relationship between HIV and GC and pTFh. In chronic treated HIV infection, lymphoid tissue is the primary site of ongoing HIV replication (58, 59). GC TFh cells have been implicated in HIV persistence by supporting viral replication during treated infection and serving as an important cellular reservoir of HIV-1 DNA.

Germinal center TFh cells are highly permissive to HIV infection, with downregulation of PD-1 during HIV-1 replication (37). Cell sorting experiments by Perreau et al. have demonstrated that TFh and CXCR5− PD-1+ populations in lymph node are most efficient in supporting HIV replication (45). A recent study looked at the role of pTFh, defined phenotypically as CD45RA− CCR7+ CXCR5+, in HIV persistence, they used an *in vitro* GFP reporter assay and found pTFh, in particular, PD1+ pTFh cells to be more permissive for HIV infection than non-pTFh cells (60).

T Follicular helper cells cells demonstrate greater HIV viral production compared to other CD4+ T-cell subsets. In untreated HIV-1 infection, CXCR5+ CD4+ T-cell subsets have been shown to contain 11- to 66-fold more HIV-1 RNA than CXCR5− subsets (37). A study in chronic HIV infection of pTFh cells demonstrated greater HIV production (measured by p24 expression after anti-CD3/anti-CD28 stimulation). Also, higher frequencies of 2-LTR circles were observed in the pTFh cells than in non-pTFh cells, confirming the idea that TFh cells support HIV persistence during ART-treated HIV infection (60).

Studies in non-human primates support the hypothesis that TFh cells in GCs act as a sanctuary site for ongoing viral replication in otherwise controlled SIV infection (61). Certain primates that can spontaneously control plasma SIV replication to levels below the limit of detection are termed “Elite” controlling monkeys, where HIV-specific CD8+-mediated viral control in extrafollicular sites was observed. Such monkeys had evidence of ongoing viral replication in GCs, from which HIV-specific CD8+ T cells are excluded (61). Bortitz et al. have published work that supports this mechanism of persistence; using samples from elite controllers, they detected viruses in lymph node with genetic and transcriptional markers of active replication most abundantly within PD1+, TFH-enriched cell populations (62). Recently, a specialized group of cytotoxic T cells that express CXCR5 have been described and termed “follicular cytotoxic T cells.” These cytotoxic cells were shown to eradicate TFh-infected cells (63). A greater understanding of this subtype will be pivotal to eradicate latently infected GC TFh cells.

## Conclusion

Strong antibody responses are pivotal to the eradication of many pathogenic infections. The importance of TFh cells in regulating B cell development and function to produce broad neutralizing antibodies is now clearly evident. TFh dysregulation in HIV infection has been well characterized. Whether these cells are preferentially infected within GCs and remain as latent reservoirs still requires further investigation. Also, whether Pre-TFh cells within secondary lymphoid tissue are targets of HIV due to their high expression of CCR5 and whether they are able to upregulate PD-1 and become fully functioning TFh cells is still unclear. Molecules targeting TFh cell function, such as PD-1 blocking antibodies and recombinant IL-21 administration are currently being used in therapeutic cancer trials. Results from these trials will determine if they would be beneficial as immunotherapy in HIV infection. However, a caveat of using such therapies in the context of HIV infection is that although it may improve TFh-B cell interactions and antibody production, immune modulation of TFh function may also increase T cell activation and lead to reactivation of the virus that may lead to an increase in additional reservoirs within GCs. Detailed studies of this important subset in terms of its function within the GCs and its relationship to other T cell and B cell subsets will shed light on possible therapeutics that may be useful in HIV infection.

## Author Contributions

JT wrote the manuscript. SF and PK edited the manuscript. JF and CP are equal contributors; both wrote and edited the manuscript.

## Conflict of Interest Statement

The authors declare that the research was conducted in the absence of any commercial or financial relationships that could be construed as a potential conflict of interest.
